# Comparative transcriptomics reveals candidate carotenoid color genes in an East African cichlid fish

**DOI:** 10.1186/s12864-020-6473-8

**Published:** 2020-01-16

**Authors:** Ehsan Pashay Ahi, Laurène A. Lecaudey, Angelika Ziegelbecker, Oliver Steiner, Ronald Glabonjat, Walter Goessler, Victoria Hois, Carina Wagner, Achim Lass, Kristina M. Sefc

**Affiliations:** 1 Institute of Biology, University of Graz, Universitätsplatz 2, A-8010, Graz, Austria; 2 Department of Comparative Physiology, Uppsala University, Norbyvägen 18A, SE-75 236 Uppsala, Sweden; 3 Department of Natural History, NTNU University Museum, Norwegian University of Science and Technology, NO-7491 Trondheim, Norway; 4 Institute of Chemistry, University of Graz, Universitätsplatz 1, A-8010, Graz, Austria; 5 Institute of Molecular Biosciences, University of Graz, Heinrichstraße 31/II, 8010, Graz, Austria; 6 BioTechMed-Graz, 8010 Graz, Austria

**Keywords:** Carotenoids, Body coloration, Color genes, Gene expression, Cichlidae, Tropheus, Lipids, RNA-Seq, BCO2

## Abstract

**Background:**

Carotenoids contribute significantly to animal body coloration, including the spectacular color pattern diversity among fishes. Fish, as other animals, derive carotenoids from their diet. Following uptake, transport and metabolic conversion, carotenoids allocated to body coloration are deposited in the chromatophore cells of the integument. The genes involved in these processes are largely unknown. Using RNA-Sequencing, we tested for differential gene expression between carotenoid-colored and white skin regions of a cichlid fish, *Tropheus duboisi* “Maswa”, to identify genes associated with carotenoid-based integumentary coloration. To control for positional gene expression differences that were independent of the presence/absence of carotenoid coloration, we conducted the same analyses in a closely related population, in which both body regions are white.

**Results:**

A larger number of genes (*n* = 50) showed higher expression in the yellow compared to the white skin tissue than vice versa (*n* = 9). Of particular interest was the elevated expression level of *bco2a* in the white skin samples, as the enzyme encoded by this gene catalyzes the cleavage of carotenoids into colorless derivatives. The set of genes with higher expression levels in the yellow region included genes involved in xanthophore formation (e.g., *pax7* and *sox10*), intracellular pigment mobilization (e.g., *tubb*, *vim*, *kif5b*), as well as uptake (e.g., *scarb1*) and storage (e.g., *plin6*) of carotenoids, and metabolic conversion of lipids and retinoids (e.g., *dgat2, pnpla2, akr1b1*, *dhrs*). Triglyceride concentrations were similar in the yellow and white skin regions. Extracts of integumentary carotenoids contained zeaxanthin, lutein and beta-cryptoxanthin as well as unidentified carotenoid structures.

**Conclusion:**

Our results suggest a role of carotenoid cleavage by Bco2 in fish integumentary coloration, analogous to previous findings in birds. The elevated expression of genes in carotenoid-rich skin regions with functions in retinol and lipid metabolism supports hypotheses concerning analogies and shared mechanisms between these metabolic pathways. Overlaps in the sets of differentially expressed genes (including *dgat2*, *bscl2*, *faxdc2* and *retsatl*) between the present study and previous, comparable studies in other fish species provide useful hints to potential carotenoid color candidate genes.

## Background

Carotenoids serve important functions in various aspects of animal life. They are physiologically important as precursors of vitamin A, as anti-oxidants as well as modulators of cell growth, gene expression and immune response [1, 2]. Moreover, given their involvement in body coloration, they function as signals in a variety of fitness-relevant contexts including mate choice, social competition and species recognition [3]. With few exceptions (e.g. [4]), animals cannot synthesize carotenoids endogenously and instead rely on the uptake of carotenoids from their diet. Notwithstanding the potential for plasticity of carotenoid-dependent body coloration [1], genetic factors play a major role in the determination of carotenoid-based patterns and hues [5]. In the skin of poikilothermic animals, carotenoids are stored in erythrophores (red pigment cells) and xanthophores (yellow pigment cells), whose formation is dependent on genes controlling fate specification of the neural crest-derived precursor cells [6–8]. The distribution of chromatophores in the integument is controlled by cellular interactions between the different types of chromatophores [9] and influenced by variation in gene expression [7, 10]. In addition to the processes involved in the spatial arrangement of chromatophores, pigmentation of xanthophores/erythrophores depends on the transport and metabolic conversions of dietary carotenoids as well as their cellular uptake and storage, all of which may be assumed to be, at least in part, under genetic control [11, 12]. The color of individual carotenoids depends on their chemical structure, in particular the number and position (within or outside end rings) of conjugated double bonds [13]. The carotenoid content of xanthophores/erythrophores typically encompasses a mixture of different carotenoids (e.g. [14], [15], which are deposited in the cells directly as derived from the diet or following endogenous metabolic conversions (e.g. [16, 17]).

**Fig. 1:**
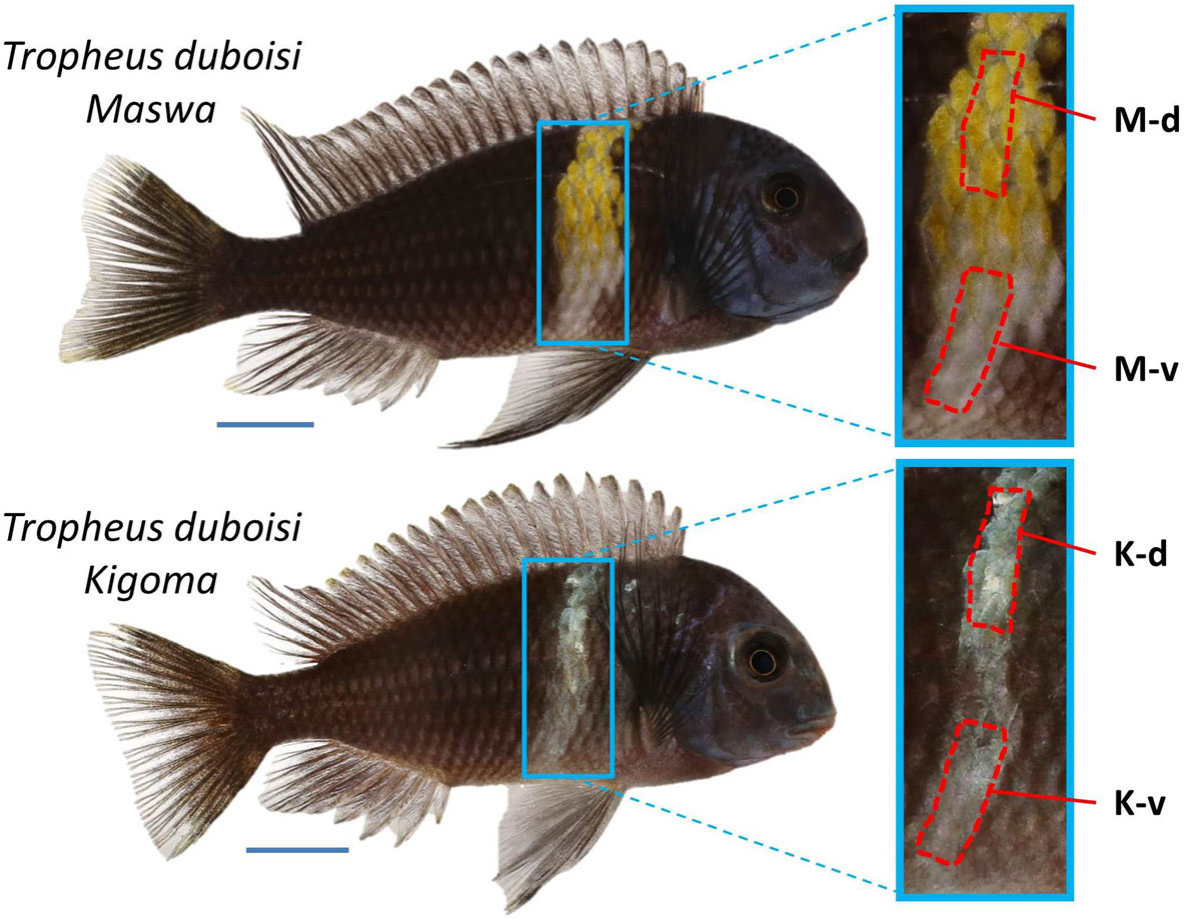
Adult males of two *Tropheus duboisi* populations used in this study. The red dashed lines specify the areas used for RNA, carotenoid and triglyceride analyses. M-d: Maswa, dorsal bar region; M-v: Maswa, ventral bar region; K-d: Kigoma, dorsal bar region, K-v: Kigoma, ventral bar region. Photographs by Wolfgang Gessl, Institute of Biology, University of Graz (www.pisces.at)

The number of genes which are known to affect carotenoid-based color diversity in vertebrates is rather small. For example, *BCO2* encodes a carotenoid-cleavage enzyme which is associated with yellow/white skin and plumage color polymorphism in birds [18, 19]; *CYP2J19* encodes a ketolase that catalyzes the metabolic conversion of dietary yellow carotenoids into red ketocarotenoids in birds and turtles [20–22]; *SCARB1* encodes a high-density lipoprotein receptor that mediates the cellular uptake of carotenoids and was found to be responsible for the presence/absence of carotenoid plumage coloration in canary breeds [23]. Comparative transcriptomic analyses revealed correlations between carotenoid-based skin color differences and the expression levels of some of the known carotenoid color genes, and identified novel candidate genes which might be involved in carotenoid-based coloration (e.g., [24–28]). In the present study, we used RNA sequencing (RNA-Seq) to test for differential gene expression associated with the presence/absence of carotenoid-based coloration in a cichlid fish. Cichlids are well known for their diversity in color patterns and hues [29], and numerous studies link cichlid carotenoid coloration to various fitness components [12]. Unlike many other poikilothermic vertebrates, in which yellow and red skin coloration is produced by mixtures of pteridine and carotenoid pigments (e.g., [30–35]), the integumentary reds and yellows of cichlids seem to be mainly, if not exclusively, produced by carotenoids [12, 14, 36, 37]. In this study, we focus on the Lake Tanganyika endemic *Tropheus duboisi*, which is characterized by a black body with one light-colored vertical bar. The color of the bar varies from white to yellow between populations. Here, we use adults of *T. duboisi* “Maswa” to compare gene expression levels in the dorsal, yellow colored region of the bar with the ventral, white colored region of the bar (Fig. 1). We also tested for differential gene expression between the same body regions in another population, *T. duboisi* “Kigoma”, which show a completely white bar without any perceptible carotenoid pigmentation (Fig. 1). The two color variants of *T. duboisi* are closely related (identical COI sequences; net distance of *p* = 0.001 in mitochondrial control region; Additional file 1, [38–40]). Dorsoventral gene expression differences that were detected in both populations were considered independent of presence/absence of carotenoid coloration, but likely reflect positional gene expression differences. The set of genes, which were differentially expressed only in the comparison between yellow and white skin of *T. duboisi* “Maswa”, included known pigmentation genes as well as genes coding for proteins involved in lipid metabolism and organelle transport.

## Results

### Transcriptome assembly

The Trinity de novo assembler generated 224,791 contigs (transcripts) and 114,215 unigenes (isoform clusters). The average length of contigs was 1178 bp, the minimum contig length was 201 bp and the longest contig was 15,959 bp. The N50 was 2297 bp, which represent 50% of the total assembles sequences having at least this contig length. The GC content was 46.11%. In total, 99.44% of the reads were assembled. The BUSCO score of the assembled transcriptome was C:82.0% [S:35.6%, D:46.4%], F:7.2%, M:10.8%, n:4584.

### Differential gene expression in the RNA-Seq experiment

**Fig. 2:**
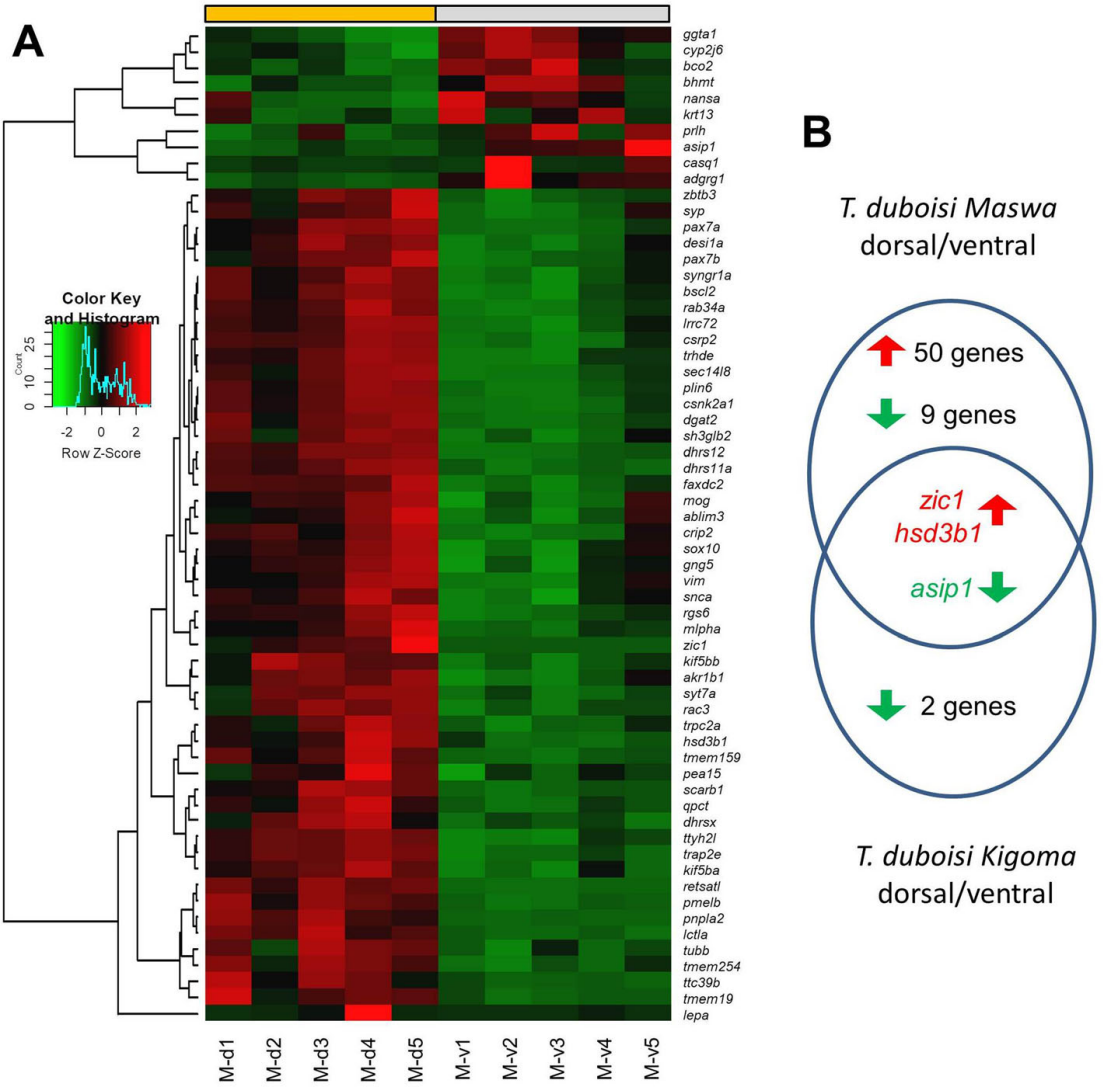
Differential gene expression. **a** Heatmap showing differential gene expression between yellow (dorsal; M-d1 – M-d5) and white (ventral; M-v1 – M-v5) skin samples of *T.duboisi* Maswa. Red and green shadings represent higher and lower relative expression levels, respectively. **b** A Venn diagram showing the numbers of differentially expressed genes in the two populations. Only three genes, *hsd3b1*, *zic1* and *asip1*, were differentially expressed in both populations

We identified a total of 62 genes with differential expression (DE) between the dorsal yellow and the ventral white skin tissue of *T. duboisi* “Maswa” (Fig. 2; Additional file 2). Three of these genes were also differentially expressed in comparison between corresponding dorsal and ventral skin regions of the entirely white-colored bar of *T. duboisi* “Kigoma”. Specifically, in both populations, expression of *asip1* was higher in the ventral than in the dorsal region, whereas expression levels of *zic1* and *hsd3b1* were higher in the dorsal regions (Fig. 2). DE of these genes in both populations suggests that these differences are unrelated to the presence (dorsal) or absence (ventral) of yellow coloration in *T. duboisi* “Maswa”. In contrast, the remaining 59 genes, which showed dorsoventral expression differences only in *T. duboisi* “Maswa”, may include genes that are associated with the presence and absence of carotenoid-based skin coloration. A large proportion of these genes (*n* = 50) showed higher expression levels in the yellow colored skin, while only nine genes were more strongly expressed in the white region (Fig. 2). Among the latter group, we highlight the elevated expression of *bco2a* (beta-carotene oxygenase 2a), coding for a carotenoid cleavage enzyme, in the white relative to the yellow colored skin tissue. Higher expression levels of genes in the yellow relative to the white skin area are expected to be, at least in part, related to the presence of carotenoid-based skin coloration. The list of genes with higher expression in the yellow skin includes transcription factors known to be involved in xanthophore formation (*pax7* and *sox10*), as well as genes which might be involved in the uptake (e.g., *scarb1*), storage (e.g., *plin6*) and metabolic conversion of carotenoids (e.g., *dgat2, pnpla2*) or in intracellular pigment mobilization (e.g., *tubb*, *vim*, *kif5b*).

**Fig. 3:**
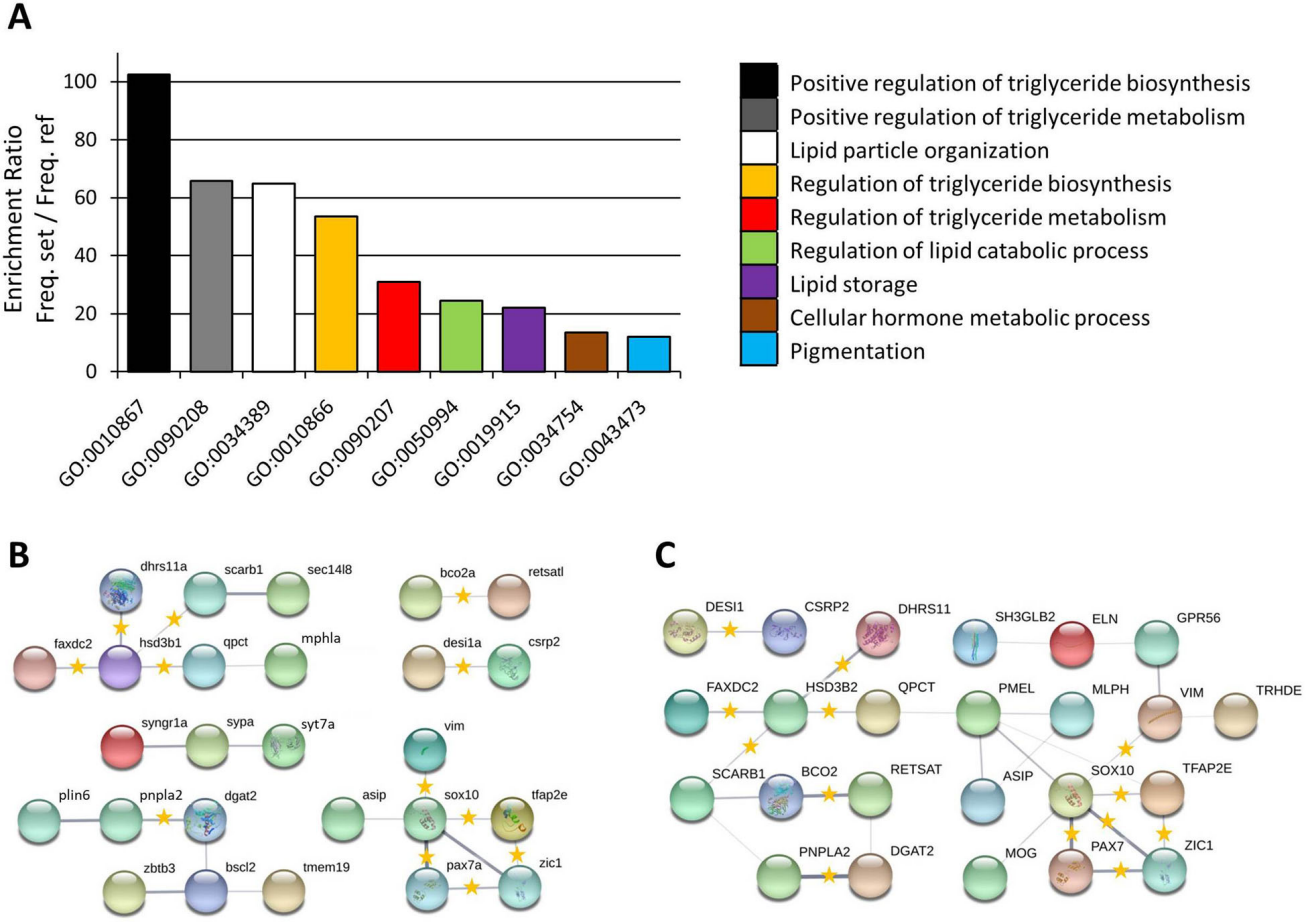
Functional enrichment and functional associations among differentially expressed genes. **a** Gene ontology enrichment analysis (Manteia) for biological processes in the differentially expressed genes (*T. duboisi* Maswa). **b** and **c** Predicted functional associations between the differentially expressed genes (both variants of *T. duboisi*) based on zebrafish **b** and chicken **c** databases in STRING v10 (http://string-db.org/)

Using the DE genes identified exclusively in *T. duboisi* “Maswa”, we tested for enrichment of gene ontology categories (biological process) relative to the zebrafish transcriptome. Enriched GO terms were associated with lipid metabolism and storage, pigmentation and hormone metabolism (Fig. 3a). Genes in the enriched GO term “cellular hormone metabolic process” were also assigned to “lipid metabolic process”. Hence, all of the DE genes assigned to enriched GO terms were associated with either lipid metabolism and storage or pigmentation.

We explored potential interactions between the proteins expressed by the DE genes using protein interactome databases of zebrafish and chicken [41], two vertebrates with integumentary carotenoid-based coloration. The interactome reconstructions (Fig. 3b and c) suggested functional connections between proteins involved in carotenoid and lipid metabolism (Bco2, Retsatl; Plin6, Pnpla2, Dgat2, Bscl2; Sec14l8, Scarb1, Hsd3b1, Faxdc2, Dhrs11) and linked the transcription factors Sox10, Pax7a, Tfap2e and Zic1 with pigmentation-associated proteins like agouti signaling protein (Asip), premelanosome protein (Pmel), melanophilin (Mlph) and vimentin (Vim). Some of the functional associations were supported by both databases and may represent ancestral, conserved molecular mechanisms of body coloration.

### Validation of transcriptome data by qPCR

**Fig. 4:**
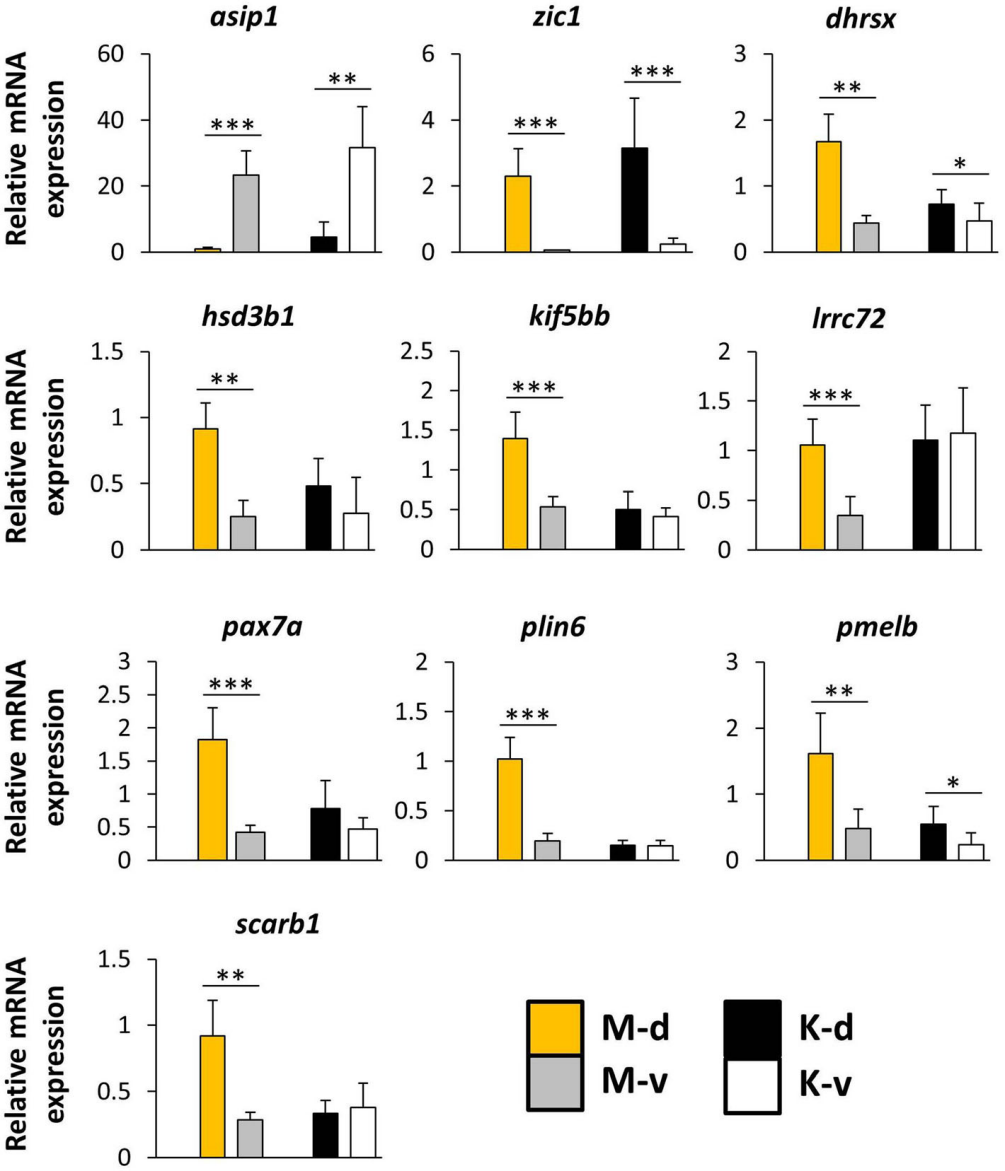
Validation of RNA-Seq expression patterns using qPCR for 10 selected genes. Bars represent means and standard deviations of RQ in three biological replicates for each skin region and population (M-d: Maswa, dorsal bar region; M-v: Maswa, ventral bar region; K-d: Kigoma, dorsal bar region, K-v: Kigoma, ventral bar region). Asterisks indicate significant differences in expression levels between the dorsal and ventral samples in within-population comparisons (paired *t*-tests; ***, *p* < 0.001; **, *p* < 0.01; *, *p* < 0.05)

In order to assess the reliability of the RNA sequencing approach, we conducted qPCR based profiling of gene expression in the dorsal and ventral bar regions of *T. duboisi* “Maswa” and *T. duboisi* “Kigoma” for ten of the DE genes from the RNA-Seq experiment (Fig. 4). Seventeen of the 20 qPCR-based tests for DE (85%) yielded results that were consistent with the RNA-Seq experiment and showed DE between skin regions in both populations (*asip1*, *zic1*) or only in the “Maswa” population (*kif5bb*, *lrrc72*, *pax7a*, *plin6*, *scarb1*). The three inconsistencies between the qPCR vs the RNA sequencing gene expression profile were observed in the white-barred “Kigoma” population, namely the lack of statistically significant higher expression of *hsd3b1* in the dorsal region and the presence of small, but statistically significant expression differences for *dhrsx* and *pmelb* (Fig. 4).

### Carotenoid and triglyceride content

Reversed-phase high performance liquid chromatography using ultraviolet and visible light detection (HPLC-UV/VIS) and liquid chromatography high resolution tandem mass spectrometry (LC-MS/MS) of skin extracts revealed the presence of both free and esterified carotenoids in the yellow colored skin of *T. duboisi* “Maswa” (Additional file 3: Figure S1). Zeaxanthin, lutein and beta-cryptoxanthin were identified by comparison of retention times, UV/VIS and high resolution mass spectra including MS/MS with carotenoid standards. Two high-abundant signals eluting at 5.05 and 5.10 min did not match any of the used carotenoid standards, but their UV/VIS spectra as well as formulas predicted from high resolution MS data (m/z [M + H]^+^ of 565.4020 corresponding to C_40_H_52_O_2_ with Δm < 5 ppm and m/z [M + H]^+^ of 567.4156 corresponding to C_40_H_54_O_2_ with Δm < 5 ppm) suggested a carotenoid structure. Additional minor peaks were considered to represent lutein-like structures based on their mass and fragmentation patterns in MS/MS experiments compared to the lutein standard. We also detected minor signals of free carotenoids, mainly zeaxanthin, in the white skin samples of *T. duboisi* “Maswa”, but no carotenoid esters (Additional file 3: Figure S1).

The concentration of triglyceride (TG), determined enzymatically, did not differ between the yellow and white skin regions of *T. duboisi* “Maswa” (mean ± s.d. = 6.36 ± 3.80 nmol TG/mg across yellow and white skin samples from 6 fish; mean difference between yellow and white region = 0.78 nmol TG/mg; t = 1.74, df = 5, *p* = 0.14 in paired *t*-test). For qualitative assessment of neutral lipid content of skin extracts, we subjected extracts to thin-layer chromatography. Spots comigrating with triolein and free cholesterol standards were visible in all skin extracts. The intensities of spots for triglyceride varied strongly between skin samples from different fish, while those for free cholesterol were similar across all samples (Additional file 3: Figure S2).

## Discussion

The transcriptome comparison between carotenoid-colored and white skin regions of the cichlid fish *T. duboisi* “Maswa” identified a set of 59 DE genes, many of which are associated with triglyceride metabolism and lipid storage or have known functions in pigmentation and retinol metabolism. Triglyceride concentrations were similar in the yellow and white skin regions, but carotenoid content differed in composition and concentration. In the following, we discuss the DE genes which might be linked to the different cellular and metabolic processes involved in carotenoid-based skin coloration.

Our study also identified genes, which were differentially expressed between dorsal and ventral skin samples of both *T. duboisi* populations, that is, probably independent of a carotenoid-based color pattern. Among those, *asip1* has an evolutionarily conserved role in the dorsoventral melanin patterning of vertebrates [42] and *zic1* determines dorsal characteristics of trunk and fin in teleost fish [43].

### Elevated expression of beta-carotene oxygenase 2a in the white skin region

The beta-carotene oxygenase BCO2 catalyzes the oxidative cleavage of yellow and red C-40 carotenoids into colorless derivatives. The elevated expression of *bco2a* in the white skin region of *T. duboisi* “Maswa” is in accordance with the absence of visible carotenoid-based coloration compared to the yellow skin region. In mammals, nonsense mutations of *BCO2* result in increased carotenoid levels and affect the color of cow milk [44] and of the adipose tissue of sheep [45]. Similarly, flesh pigmentation of salmon is associated with the fish-specific *BCO2-like* gene [46]. In birds, the *BCO2*-containing region was a differentiation outlier in a genome scan comparison between wood warblers varying in carotenoid-based plumage coloration [19], and expression differences of *BCO2* in domestic chicken correlate with a yellow-white skin polymorphism [18]. Our data suggest that similar effects of Bco2 on carotenoid-based integumentary color polymorphism exist in fish. Importantly, the substrate specificity of BCO2 (determined using chicken BCO2, [47]) includes xanthophylls (beta-cryptoxanthin, lutein and zeaxanthin), which we identified in the skin of *T. duboisi* “Maswa”.

### Xanthophore specification and differentiation

Chromatophores emanate from neural crest-derived precursor cells. Their specification and differentiation into various pigment cell types is controlled by genetic factors and associated with cell type specific gene expression profiles [48, 49]. Among the transcriptional regulators with elevated expression in the yellow bar region of *T. duboisi* “Maswa” was a member of SRY-related HMG-box family, *sox10*, which is well known for its pivotal role in fate specification of chromatophores in fish [50–52]. In zebrafish, Sox10 is required for the development of all pigment cells except leucophores, and in medaka, cooperative interaction between transcription factors, encoded by *sox5* and *sox10*, is essential to promote xanthophore fate and to repress leucophore fate [52]. Moreover, in zebrafish, xanthophore formation depends quantitatively on the number of functional alleles of the *sox10* paralogs [52]. The increased expression of *sox10* in the yellow bar region of *T. duboisi* “Maswa” suggests that this region is an active site for fate specification of xanthophores.

Another transcription factor gene with increased expression in the yellow bar region was *pax7* (particularly, both teleost specific paralogs, *pax7a* and *pax7b*), a member of the paired box (PAX) family which is essential for xanthophore formation in zebrafish and medaka [6–8]. Variance in *pax7* expression correlates with xanthophore and melanophore based body color patterning in a group of cichlid fish [10], and both *pax7* and *sox10* are differentially expressed in xanthophore-rich versus unpigmented fin tissues of cichlids and guppies [25, 28].

### Carotenoid, lipid and retinol metabolism

Fish, like other animals, acquire carotenoids from their diet. Dietary carotenoid esters are hydrolyzed before absorption and transported in the blood circulation together with fatty acids with the aid of lipoproteins [53]. Uptake of carotenoids into target tissue cells is mediated by proteins and may be selective [53]. The skin of fish contains mixtures of dietary and converted carotenoids, which are often esterified with fatty acids [54]. Within xanthophore and erythrophore cells, the lipophilic carotenoids are concentrated in carotenoid droplets, special organelles with structural similarities to the well-studied lipid droplets [55], which consist of a core of neutral lipids surrounded by a phospholipid monolayer and embedded proteins [56]. Esterification of hydroxylated carotenoids increases their stability and liposolubility [54] and may be important for carotenoid droplet formation [55].

Evidence for associations with the uptake and storage of integumentary carotenoids exists for two of the genes, which were overexpressed in the yellow relative to the white skin of *T. duboisi* “Maswa”. One is *scarb1*, which codes for a lipoprotein receptor essential for the cellular uptake of carotenoids across a range of vertebrates and invertebrates [5]. Abnormal splicing of the gene results in reduced carotenoid uptake and a loss of carotenoid-based plumage coloration in a canary mutant [23], while the presence of a *scarb1* paralog was associated with flesh pigmentation in the Atlantic salmon [53]. Importantly, a recent study demonstrated that *scarb1* is required for the deposition of carotenoids into adult xanthophores of the zebrafish [57] and expression levels of *scarb1* co-varied with skin carotenoid content in a lizard [26].

The second gene with a known function in carotenoid skin pigmentation is *plin6*, a teleost perilipin gene, which is highly expressed in zebrafish xanthophores and targeted to the surface of carotenoid droplets [55]. Knockout of *plin6* led to severe reductions of integumentary carotenoid and triglyceride levels and interfered with the intracellular aggregation of carotenoid droplets [55].

Several additional DE genes of this study (*bscl2*, *dgat2*, *lepa*, *pnpla2*) have known functions in lipid storage and metabolism and may be linked to yellow coloration via the homologies between carotenoid and fat storage in the skin of fish. Since the concentrations of triglycerides were similar in the white and yellow skin regions, the observed expression level differences of these genes cannot be explained by a gradient in skin fat content, but may instead be directly related to skin carotenoids. Seipin (encoded by *bscl2*) regulates lipid droplet formation [58] and was recently suggested as a xanthophore marker protein based on its expression in zebrafish xanthophores [57]. Indeed, seipin showed elevated expression in the carotenoid-colored skins of guppies and clownfish [28, 59], consistent with our finding in *T. duboisi*. The acyltransferase encoded by *dgat2* is associated with lipid droplets and catalyzes the esterification of diacylglycerol [56]. Consistent with our data, correlations between *dgat2* expression and integumentary carotenoids in guppies [28] and in a lizard species [26] support a connection between *dgat2* expression and carotenoid-based skin coloration. Notably, expression differences of *dgat2* in our experiment coincide with the presence and absence of carotenoid esters in the yellow and white skin, respectively. Another DE gene, *pnpla2*, codes for adipose triglyceride lipase (ATGL), which catalyzes the hydrolysis of triglyceride esters into diacylglycerol and a fatty acid [60, 61] as well as the hydrolysis of retinyl esters [62]. Our data possibly indicates an analogous role of ATGL in the mobilization of carotenoid stores. The role of leptin (encoded by *lepa*) in the lipid metabolism of fish is not entirely clear, but may consist in the promotion of lipolysis [63]. Our DE gene set also included two lipoprotein coding genes, *ttc39b* and *sec 14 l8*. Intriguingly, *TTC39B* has recently been found to be located in a genomic region co-segregating with an avian carotenoid-based color polymorphism [64].

Dietary carotenoids undergo various metabolic conversions, but the responsible enzymes remain largely unknown [5, 12]. One of our DE genes, *faxdc2*, belongs to the fatty acid hydroxylase superfamily, members of which have been shown to catalyze the hydroxylation of carotenoids in plants, notably the conversion of beta-carotene to zeaxanthin [65–67]. Additional DE genes are associated with the metabolism of retinoids, i.e. carotenoid derivatives, and include three *dhrs* genes (*dhrs11a*, *dhrs12* and *dhrsx*), *akr1b1* (an aldo-ketoreductase with retinaldehyd reductase activity, [68]) and *retsatl*. Enzymes of the short chain dehydrogenase/reductase SDR family (*dhrs*) catalyze the reversible oxidation/reduction of retinol and retinal [69]. Although their retinoid redox activity might suggest a function in the enzymatic conversion of carotenoids, *dhrsx* also deserves attention due to its association with xanthophore organization in the fins of cichlids and zebrafish [70, 71]. We note, however, that qPCR detected dorsally elevated expression of *dhrsx* also in the white-barred population of *T. duboisi*, suggesting that the dorsoventral expression differences of *dhrsx* are not necessarily connected with carotenoids in the yellow-barred population. *Retsatl* encodes for a retinol saturase like protein of unknown activity in zebrafish [72], while the avian retinol saturase (RETSAT) was recently shown to catalyze the saturation of apo-carotenes in retinal cells [73]. The latter study [73] provides two examples for new roles of retinoid metabolizing enzymes (RETSAT and retinol dehydrogenase RDH12) in the conversion of additional carotenoid substrates, fuelling hopes that some of the elusive carotenoid color genes may be identified among the better-studied set of retinoid metabolism genes (see also [74]). Indeed, genes coding for retinoid metabolizing enzymes were also overexpressed in the orange relative to the white skin of clownfish [59]. Congruent with our data, these include *retsatl* and *dhrs12*, and additionally, *rdh12* as well as a retinaldehyd binding protein gene (see Table S3 in [59]).

### Physiological color change

Cichlid fish communicate via rapid physiological color changes, which are mediated by translocation of pigment within chromatophores [29, 75]. In this process, pigment granules such as carotenoid droplets associate with motor proteins and then disperse and aggregate along the cytoskeleton. Molecular mechanisms of pigment mobilization have been well studied in melanophores (e.g. [76]), and are supposed to be similar in erythrophores and xanthophores [77], although different cytoskeletal elements may be utilized [78]. Pigment mobilization is controlled by levels of Ca^2+^ and cyclic AMP, which respond to extracellular signals such as hormonal, neuronal and light stimuli [76, 77]. Dispersal and aggregation of pigment involve phosphorylation or dephosphorylation, respectively, of proteins bound to pigment granules [77], and G-protein signaling was shown to be involved in the light response of erythrophores in a cichlid fish [79].

A considerable proportion of DE genes with higher expression in the yellow-colored skin of *T. duboisi* “Maswa” can, on a hypothetical basis, be linked with pigment mobilization in xanthophores. Among these genes, some are involved in signaling, including *gng5* coding for a G-protein subunit [80], *rgs6* coding for a RGS (regulator of G-protein signaling) protein [81], *trpc2a* coding for an intracellular Ca2+ release channel [82], *csnk2a1* coding for a pleiotropic protein kinase [83], and the two GTPase genes, *rab34* and *rac3*. Several additional DE gene products may play a role in movement of carotenoid droplets along the cytoskeleton. These include the cytoskeletal genes tubulin (*tubb*) and vimentin (*vim*), which code for proteins of microtubuli and intermediate filaments, respectively, and are required for pigment granule movement in teleost melanophores [84, 85]. *ablim3* encodes a cytoskeletal actin-binding protein [86] and the two paralogs of *kif5b* code for the motor protein kinesin, which effectuates melanosome dispersal in fish melanophores [85, 87]. Furthermore, in zebrafish, melanophilin (*mlpha*) facilitates melanosome dispersal [88]. Links of *mlpha* and *vim* expression with the presence of xanthophores in fish skin are further supported by their elevated expression in orange relative to white skin of the clownfish (suppl. Table 3 in [59]). Intriguingly, both Vimentin and Rab34 are among a number of proteins associated with lipid droplets [89–91], and it was recently shown that expression of the lipid droplet-associated Plin6 protein promotes the aggregation of carotenoid droplets in zebrafish xanthophores [55].

## Conclusion

In this study, we analyzed differential gene expression related to carotenoid-based skin coloration in a cichlid fish. Expression differences were detected for genes with known functions in xanthophore formation (*pax7, sox10*) and carotenoid-based coloration of fish, such as *scarb1* and *plin6*. Our study provides support for a role of the carotenoid cleavage enzyme Bco2 in fish integumentary coloration, analogous to previous findings in birds. Additionally, we detected signals for genes involved with intracellular organelle movement, which may be related with the ability of fish xanthophores to contribute to physiological color change by aggregation and dispersal of carotenoid droplets. In the present as well as in comparable transcriptome comparisons (e.g., [26, 28, 59]), differential expression of genes that have been characterized in the context of lipid metabolism may reflect the analogies between carotenoids and neutral lipids in terms of integumentary storage and metabolic conversion. Likewise, DE enzymes involved in retinol metabolism may be hypothesized to catalyze analogous metabolic conversions of carotenoids. Overlaps in DE gene sets across different species and experiments may provide useful hints to novel carotenoid color candidate genes, such as *dgat2*, *bscl2*, *faxdc2* and *retsatl* on the basis of this and previous studies.

## Methods

### Sampling of skin tissue

The experiment employed adult, captive bred males of *T. duboisi* population “Maswa” (*n* = 12 across all experiments) and *T. duboisi* population “Kigoma” (*n* = 5 across all experiments). We note that the study species, *T. duboisi*, is listed as vulnerable in the IUCN Red List of Threatened Species. Ethical approval for the use of *T. duboisi* in our experiment was obtained from the ethics committee of the University of Graz (approval number 39/84/63 ex 2018/19).

Fish keeping and euthanasia was performed under permit BMWFW-66.007/0004-WF/V/3b/2016 issued by the Federal Ministry of Science, Research and Economy of Austria (BMWFW) in accordance with national guidelines and regulations. *T. duboisi* “Maswa” had been bred in our lab and were third-generation offspring from commercially purchased, captive-bred fish. Captive-bred *T. duboisi* “Kigoma” were purchased from Cichlidenstadel (Alerheim, Germany; https://www.cichliden-stadel.de/en/) four months before the start of the experiment. Prior to skin sampling, the fish were kept in a large, mixed-sex group and fed identical diets of flake food. Before dissection, fish were sacrificed in a solution of 2 g MS-222 per 1 L water. Skin samples of the dorsal and ventral bar region (Fig. 1) were taken by carefully pulling off the skin, separating it from underlying tissue and removing the scales. Scales were removed in order to avoid variation in skin and scale content among tissue samples.

### RNA extraction

Skin samples (n = 5 fish of each population) were immediately transferred into RNAlater (Qiagen) and stored at − 20 °C. For RNA extraction, skin samples were immersed into 250 μL of LBA buffer mixed with the recommended volume of 1-thioglycerol and 1.4 mm ceramic spheres and then homogenized using FastPrep-24 Instrument (MP Biomedicals, CA, USA). Total RNA was extracted from the tissue samples using the ReliaPrep™ RNA Tissue Miniprep System Kit (Promega) according to the manufacturer’s protocol for fibrous tissues. RNA was eluted in 30 μL nuclease-free water, quantified with a Nanophotometer (IMPLEN GmbH, Munich, Germany) and analyzed for quality on a R6K ScreenTape System using an Agilent 2200 TapeStation (Agilent Technologies). All samples achieved RNA integrity numbers (RIN) above 7.

### RNA-Seq library preparation, de novo assembly and expression analysis

Library preparation was performed according to the protocol of the Standard TruSeq Stranded mRNA Sample Prep Kit (Illumina) using 1500 ng RNA from each sample. We tested the quality of the libraries using D1000 ScreenTapes on a TapeStation 2200 (Agilent Technologies). Sequencing was performed in the NGS Facility at Vienna Biocenter Core Facilities (VBCF, Austria) on one lane of an Illumina HiSeq2500 flowcell, which generated 125 bp paired-end reads (3.4–14.1 million raw reads per sample; Additional file 3: Table S2). The demultiplexing of the raw reads was performed by the sequencing facility based on unique barcodes introduced in each sample during library preparation. A quality control step was conducted on the raw reads of each sample through the FASTQC tool [92]. The low quality reads for each sample were removed based on a recommended standard quality trimming step using Trimmomatic software [93]. Only reads with a phred + 33 quality score of at least 34 for all bases, and a minimum length of 50 bp were retained for downstream analyses (3.3–13.9 million trimmed reads per sample; Additional file 3: Table S2). Sequence reads are available from the NCBI sequence read archive (SRA) under the accession number PRJNA540663. Given the absence of genetic divergence between the two color variants of *T. duboisi* (Additional file 1), the de novo transcriptome assembly of the bar skin region of *T. duboisi* was performed based on the quality trimmed paired-end reads of all 20 samples of both variants. We used the Trinity software package with default parameters [94, 95], which assembled the high-quality reads into contigs. The Trinity package consist of modules, which assemble and cluster contigs and finally reconstruct the transcripts and isoforms in their final form (detailed process described in [95]). The quality of the transcriptome assembly obtained from Trinity was subsequently assessed using the Trinity tookit design to generate standard metrics and N50 statistics. Additionally, the percentage of reads assembled was computed using bowtie2 v2.2.9 [96] and the transcriptome completeness was estimated using BUSCO v3.0.2 [97, 98] with the “actinopterygii” lineage.

Based on the transcriptome assembly, the transcript abundances were quantified for each sample using Kallisto, a tool integrated in the Trinity software package, in order to obtain sample-specific expression levels of each transcript at gene level [99]. Transcripts per million transcripts (TPM), generated by Kallisto, was used as gene expression unit for downstream analysis. To normalize the data across samples, the weighted trimmed mean of the log expression ratios (TMM) was used. For each of the two *T. duboisi* populations, gene expression levels were then compared between the dorsal and ventral parts of the bar region. We used Trinity to construct normalized expression matrices of all samples and the edgeR package, from the R Bioconductor software (R version 3.4.4, R Development Core Team 2018), to detect differentially expressed transcripts [100–103]. Significantly differentially expressed genes were extracted from the results, using a false-discovery rate (FDR) cutoff of 0.1 [104] and a minimum of 1 fold-change (two times relative expression difference).

To annotate the genes, we first used TransDecoder software (http://transdecoder.github.io) to identify ORFs with complete coding sequences. TransDecoder identifies candidate protein-coding regions based on nucleotide composition through detection of a minimum length ORF per gene, computation of a log-likelihood score for each ORF and reporting the longest ORFs per gene [95]. To maximize sensitivity for capturing ORFs with functional significance, we scanned all ORFs for homology through BLAST tool [105] against coding sequences (CDS) of Nile tilapia and two other distant teleost fish species *Danio rerio* and *Gasterosteus aculeatus* for further confirmation. We used the following NCBI CDS annotations: assembly ASM185804v2 (ID: GCF_001858045.1) for the Nile tilapia, assembly GRCz10 (ID: GCF_000002035.5) for *Danio rerio* and assembly CriGri_1.0 (ID: GCF_000223135.1) for *Gasterosteus aculeatus*.

The gene ontology (GO) term analysis for biological processes was conducted with Manteia, a free online tool for data mining of vertebrate genes and included the 59 genes that were differentially expressed in *T. duboisi* “Maswa” (but not “Kigoma”). Enrichment criteria were set to FDR < 0.01 and a minimum of 2 genes in each GO term [106]. The knowledge based interactions between the gene products (64 DE genes from both populations) were explored by STRING v10 (http://string-db.org/) using zebrafish and chicken databases for protein interactomes [41].

### Real time quantitative PCR (qPCR)

Candidate reference genes were selected from the transcriptome data as follows [107, 108]. In each transcriptome comparison (*T. duboisi* “Maswa” and *T. duboisi* “Kigoma”), we identified the genes with no expression difference (FDR = 1) between dorsal and ventral skin tissue and ranked them according to their expression level to obtain the top 200 genes with highest expression. We next ranked these genes by their coefficient of variation (CV of expression levels) across biological replicates. The top 10 genes shared between both transcriptome comparisons were chosen as candidate reference genes (Additional file 4). Five of the candidate genes encode ribosomal proteins, four were members of the large ribosomal subunit (*rpl3, rpl8, rpl30* and *rpl34*) and one belonged to the group of small ribosomal subunits (*rps17*). Based on qPCR experiments (see below), the ten candidate reference genes were ranked according to expression stability by three different algorithms: BestKeeper [109], NormFinder [110] and geNorm [111]. According to BestKeeper SD ranking, two of the ribosomal protein genes, *rpl34* and *rpl8*, displayed high variation across all the skin tissues (SD > 1.5) and were discarded from further consideration. All three algorithms ranked *eif5a* and *rps17* as the first and second most stable reference genes, respectively (Table 1). Hence, we used the geometric means of the Cq values of *eif5a* and *rps17* (Additional file 4) to normalize Cq values of target genes in each sample (ΔCq _target_ = Cq _target_ – Cq _reference_).

**Table 1:** Ranking and statistical analyses of candidate reference genes in the skin samples from *Tropheus duboisi*. The selected reference genes *(rps17*, *eif5a*) are highlighted by grey shading. According to BestKeeper SD ranking, *rpl34* and *rpl8* displayed high variation across all the skin tissues (SD > 1.5) and were discarded from further consideration

BestKeeper				geNorm		NormFinder	
Ranks	SD	Ranks	r	Ranks	M	Ranks	SV
*rpl3*	0.958	*eif5a*	0.989	*eif5a*	0.404	*eif5a*	0.113
*rps17*	0.981	*rps17*	0.986	*rps17*	0.406	*rps17*	0.152
*pabpc1b*	0.997	*eef1a1l1*	0.984	*rpl3*	0.425	*pabpc1b*	0.154
*rpl30*	1.046	*hspa8*	0.983	*pabpc1b*	0.442	*eef1a1l1*	0.169
*eif5a*	1.128	*rpl3*	0.983	*eef1a1l1*	0.442	*rpl3*	0.183
*eef1a1l1*	1.171	*pabpc1b*	0.978	*rpl30*	0.484	*rpl30*	0.190
*ppiab*	1.175	*rpl30*	0.938	*hspa8*	0.486	*hspa8*	0.211
*hspa8*	1.227	*ppiab*	0.927	*ppiab*	0.659	*ppiab*	0.257
*rpl34*	1.669	*rpl34*	–	*rpl34*	–	*rpl34*	–
*rpl8*	1.850	*rpl8*	–	*rpl8*	–	*rpl8*	–

Abbreviations: *SD* Standard deviation, *SV* Stability value, *M* Average expression stability value

To design qPCR primers for candidate reference genes and a subset of the differentially expressed genes from the RNA-Seq approach, we constructed an alignment of the assembled sequence of each gene and its homologous sequences in other African cichlids including three species of the tribe Tilapiini (*O. aureus*, *O. mossambicus* and *O. niloticus*), one species of Lamprologini (*Neolamprologus brichardi*), one species of Ectodini (*Callochromis macrops*), and four species of Haplochromini (*Maylandia zebra*, *Pundamilia nyererei*, *Ctenochromis horei* and *Astatotilapia burtoni*) [25, 112, 113]. This allowed us to find conserved sequence regions across the species and at the exon junctions (using CLC Genomic Workbench, CLC Bio, Denmark and the annotated genome of *Oreochromis niloticus* from the Ensembl database, http://www.ensembl.org). All primers were designed to produce short amplicons (< 250 bp) using Primer Express 3.0 (Applied Biosystems, CA, USA) and their structural configurations were evaluated by OligoAnalyzer 3.1 (Integrated DNA Technology) (Additional file 4).

500 ng of RNA of each skin sample extract were used for first strand cDNA synthesis using the High Capacity cDNA Reverse Transcription kit (Applied Biosystems) according to the manufacturer’s protocol. cDNA was diluted 1 + 3 (v/v) for the subsequent qPCR reactions, which followed the protocol provided by Maxima SYBR Green/ROX qPCR Master Mix (2X) (Thermo Fisher Scientific, Germany) and the guidelines for optimal experimental set-up [114]. The qPCR program started with a 2 min hold at 50 °C, a 10 min hold at 95 °C, followed by 40 cycles of 15 s at 95 °C and 1 min at 60 °C, and finally a dissociation step at 60 °C – 95 °C. The primer efficiencies (E values) were calculated in LinRegPCR v11.0 [115] (Additional file 4).

For each gene, the normalized expression in one sample from the dorsal bar region of *T. duboisi “Maswa”* was set as calibrator for the calculation of ΔΔCq values (ΔCq _target_ – ΔCq _calibrator_) and relative expression levels (RQ) were determined by the 2^−ΔΔCq^ method [116]. The log-transformed RQ values were used for paired *t*-tests to infer statistically significant differences between the skin bar regions.

### Extraction and analysis of integumentary carotenoids

Fish were kept and fed as described above. Two adult male *T. duboisi* “Maswa” were sacrificed in a solution of 2 g/L MS-222. Skin samples were taken from the yellow and the white bar region as described for RNA analysis and extracted over night at 4 °C in 150 μL solution of acetone with butylated hydroxytoluene (BHT, 1 g/L). The corresponding extracts of the two individuals were pooled to attain sufficient concentrations of carotenoids for reversed-phase high performance liquid chromatography (HPLC) analysis. Carotenoid standards (lutein, tunaxanthin, β-carotene, (3R,3’R)-zeaxanthin, (rac.)-α-carotene, β-cryptoxanthin, canthaxanthin, astacene, (rac./meso)-astaxanthin, rhodoxanthin) were obtained from CaroteNature GmbH (Lupsingen, Switzerland). Initial HPLC was carried out in the Agilent 1290 UHPLC System equipped with an Agilent Zorbax Eclipse Plus C18 column (2.1 × 150 mm, 1.8 μm Rapid resolution HD, Agilent Technologies, Santa Clara, US). Mobile phase A consisted of acetonitrile (gradient grade, VWR International, UK), water (18.2 MΩ cm, obtained from a Millipore Milli-Q reference ultrapure water purification system, USA) and formic acid (MS grade, Merck, Germany) in a ratio of 80 + 20 + 0.1 v/v; mobile phase B consisted of 2-propanol (HPLC grade, VWR International, UK). A gradient with the following settings was used at a flow rate of 0.49 mL/min: 0 to 13.5 min 0% B to 100% B, 13.5 to 15 min 100% B, three minutes equilibration with 0% B. An injection volume of 1 μL was used and the column temperature was set to 55 °C. The detector obtained UV/VIS spectra (210–640 nm, 2 nm steps), and wavelengths of 440 and 480 nm with a bandwidth of 8 nm and a sampling rate of 40 Hz were recorded.

For mass spectrometric analyses, carotenoids were separated (column as above) on a Dionex Ultimate 3000 UHPLC System (Thermo Scientific, Waltham, USA) connected to a Q-Exactive Orbitrap mass spectrometer equipped with an electrospray ionization source (Thermo Scientific, Waltham, USA). A gradient with the following settings was used at a flow rate of 0.5 mL/min: 0 to 13.5 min 0% B to 90% B, 13.5 to 20 min 90% B, from 20 to 20.5 min equilibration from 90% B to 0% B and which was then kept for 4.5 min. The injection volume was 10 μL.

MS/MS experiments were performed in positive mode with spray voltages of + 3.5 kV. Nitrogen was used as drying gas at a temperature of 350 °C; sheath gas flow was set to 50 instrument units (IU) and auxiliary gas flow was 12.5 IU; capillary temperature was set to 265 °C. The full MS scan range was set to 250–900 m/z at a resolution of 70,000 (full width at half maximum (FWHM), specified at m/z 200) with data-depending MS/MS of the five most abundant signals per scan event. The fragmentation with combined normalized collision energies of 20, 40 and 60 was performed at a resolution of 35,000 (FWHM).

### Extraction and analysis of integumentary triglycerides (TG)

Skin samples of yellow and white skin regions (approx. 20 mg) were excised from six adult males of *T. duboisi* “Maswa” as described for RNA analysis. Lipids were extracted over night at 4 °C in 150 μl acetone, containing 1 g/L butylated hydroxytoluene as antioxidant. Lipid extracts were brought to dryness and suspended in 0.1% Triton X-100. Lipids were solubilized by sonication using a water bath sonicator (Transsonic T460, Schmidbauer KG, Singen, Germany) for 5 min, followed by incubation at 37 °C under constant shaking (450 rpm) for 10 min. TG concentrations were determined colorimetrically using Infinity™ Triglyceride Liquid Stable Reagent (Thermo Fischer Scientific, Waltham, MA) and glycerol as standard. The free glycerol content of lipid extracts was typically less than 1 ‰. The amounts of TG were calculated and normalized to wet tissue weight. Differences in TG content between white and yellow skin samples were tested by paired *t*-tests.

For thin-layer chromatography (TLC), acetone lipid extracts were spotted on a silica gel 60 (Merck, Darmstadt, Germany). For comparison, standard solutions containing triolein or cholesterol (Sigma-Aldrich, St. Louis, MI) were applied. The silica gel was developed using n-hexane/diethylether/acetic acid (70/29/1, v/v/v) as solvent system. Lipids were visualized by charring using a solution containing 10% copper (II) sulfate and 10% phosphoric acid, followed by incubation at 120 °C for 20 min.

## Supplementary information

**Additional file 1. **Supplementary information on mtDNA sequence similarity between white-bar (Kigoma type) and yellow-bar (Maswa-type) *T. duboisi*.

**Additional file 2: Table S1.** Differentially expressed genes identified in the RNA-Seq experiment.

**Additional file 3: Table S2.** Number of RNA sequencing reads obtained for each sample. **Figure S1.** HPLC chromatograms of skin extracts. **Figure S2.** Neutral lipid analysis by thin-layer chromatography (TLC).

**Additional file 4. **Supplementary information on the qPCR experiment: Candidate reference genes selected based on RNA-Seq data; qPCR primers for candidate reference and target genes; Cq and RQ values of target genes; paired *t*-tests for dorsoventral expression level differences.

## Data Availability

The datasets supporting the conclusions of this article are available as follows: Sequence reads from the RNA-Seq experiment are available from the NCBI sequence read archive under the accession number PRJNA540663 (https://www.ncbi.nlm.nih.gov/bioproject/540663) [117]. Data from the qPCR experiment are provided in the supplementary information (Additional file 4).

## Abbreviations

ABLIM3: Actin binding LIM protein family, member 3
AKR1B1: Aldo-keto reductase family 1, member B1 (aldose reductase)
ASIP1: Agouti signalling protein 1
ATGL: Adipose triglyceride lipase
BCO2: Beta-carotene oxygenase 2
BHT: Butylated hydroxytoluene
BP: Base pairs
BSCL2: Berardinelli-Seip congenital lipodystrophy 2 (seipin)
C: Carbon
Ca: Calcium
cDNA: Complementary DNA
Cq: Quantification cycle
CSNK2A1: Casein kinase 2, alpha 1 polypeptide
CV: Coefficient of variation
CYP2J19: Cytochrome P450 family member 2 J19
DE: Differential expression; differentially expressed
DGAT2: Diacylglycerol O-acyltransferase 2
DHRS: Dehydrogenase/reductase SDR family
DHRS11: Dehydrogenase/reductase (SDR family) member 11
DHRS12: Dehydrogenase/reductase (SDR family) member 12
DHRSX: Dehydrogenase/reductase (SDR family) X-linked
E values: Primer efficiency values
EIF5A: Eukaryotic translation initiation factor 5A
FAXDC2: Atty acid hydroxylase domain containing 2
FDR: False discovery rate
GNG5: Guanine nucleotide binding protein (G protein), gamma 5
GO: Gene ontology
G-protein: Guanine nucleotide-binding protein
GTPase: Guanosin triphosphate hydrolyizing enzyme
HPLC: High performance liquid chromatography
HPLC-UV/VIS: High performance liquid chromatography using ultraviolet and visible light detection
HSD3B1: Hydroxy-delta-5-steroid dehydrogenase, 3 beta- and steroid delta-isomerase 1
KIF5B: Kinesin family member 5B
LC-MS/MS: Liquid chromatography high resolution tandem mass spectrometry
LEPA: Leptin a
LRRC72: Leucine rich repeat containing 72
m/z: Mass to charge ratio
MLPH: Melanophilin a
MS/MS: Tandem mass spectrometry
MS-222: Tricaine methanesulfonate
PAX: Paired box gene
PAX7: Paired box 7
PLIN6: Perilipin 6
PMEL: Premelanosome protein
PNPLA2: Patatin-like phospholipase domain containing 2
qPCR: Quantitative polymerase chain reaction
RAB34: RAB34, member RAS oncogene family
RAC3: Rac family small GTPase 3a
RDH12: Retinol dehydrogenase 12
RETSAT: Retinol saturase (all-trans-retinol 13,14-reductase)
RETSATL: Retinol saturase (all-trans-retinol 13,14-reductase) like
RGS6: Regulator of G protein signaling 6
RIN: RNA integrity number
RNA-Seq: RNA sequencing
RPL: L ribosomal protein
RPS: S ribosomal protein
RQ: Relative expression levels
SCARB1: Scavenger receptor class B, member 1
SDR: Short chain dehydrogenase/reductase
SEC14L8: SEC14-like lipid binding 8
SOX10: SRY (sex determining region Y)-box 10
SOX5: SRY (sex determining region Y)-box 5
SRY-related HMG-box: Sex determining region Y related high mobility group box
TFAP2E: Transcription factor AP-2 epsilon
TG: Triglyceride
TLC: Thin layer chromatography
TRPC2A: Transient receptor potential cation channel, subfamily C, member 2a
TTC39B: Tetratricopeptide repeat domain 39B
TUBB: Tubulin, beta 5
UV/VIS: Ultraviolet and visible light
VIM: Vimentin
ZIC1: Zinc finger protein family member 1
Δm: Mass difference
