# Supplementary material for: Comparative transcriptomics reveals candidate carotenoid color genes in an East African cichlid fish
Source: BMC Genomics. 2020 Jan 16;21:54. doi: 10.1186/s12864-020-6473-8 (PMC6966818; doi:10.1186/s12864-020-6473-8)
Supplement: Supplementary file 1 — Additional file 1. Supplementary information on mtDNA sequence similarity between white-bar (Kigoma type) and yellow-bar (Maswa-type) T. duboisi. [file 12864_2020_6473_MOESM1_ESM.docx]

**Additional File 1**

**Comparative transcriptomics reveals candidate carotenoid color genes in an East African cichlid fish**

Ehsan Pashay Ahi, Laurène A. Lecaudey, Angelika Ziegelbecker, Oliver Steiner, Ronald Glabonjat, Walter Goessler, Victoria Hois, Carina Wagner, Achim Lass, Kristina M. Sefc

**Genetic similarity between the *T. duboisi* color variants.** White-bar (Kigoma type) and yellow-bar (Maswa-type) *T. duboisi* are closely related and therefore provide the opportunity to control for positional gene expression differences that are unrelated to the presence/absence patterns of carotenoid coloration in the corresponding skin regions. Mitochondrial COI sequences of the two color variants of *T. duboisi* are identical (656 bp). There is some variability in mitochondrial control region sequences (824 bp), but Maswa-type and Kigoma-type *T. duboisi* are polyphyletic with respect to each other and variation within and between morphs is similar (net divergence between morphs: p = 0.001). Results are based on the following sequence data (Genbank accession numbers):

| **COI** |  |
| --- | --- |
| *Maswa morph* |  |
| KF205353 | Van Steenberge et al. 2015 |
| KF205355 | Van Steenberge et al. 2015 |
| KF205357 | Van Steenberge et al. 2015 |
| KF205359 | Van Steenberge et al. 2015 |
| KU193910 | Breman et al. 2016 |
| KU193911 | Breman et al. 2016 |
| KU193912 | Breman et al. 2016 |
| *Kigoma morph* |  |
| KF205345 | Van Steenberge et al. 2015 |
| KF205347 | Van Steenberge et al. 2015 |
| KF205349 | Van Steenberge et al. 2015 |
| KF205351 | Van Steenberge et al. 2015 |
| KU193899 | Breman et al. 2016 |
| KU193913 | Breman et al. 2016 |
| KU193914 | Breman et al. 2016 |
| KU193915 | Breman et al. 2016 |
|  |  |
| **Control region** |  |
| *Maswa morph* |  |
| GQ995915 | Koblmüller et al. 2010 |
| GQ995916 | Koblmüller et al. 2010 |
| GQ995917 | Koblmüller et al. 2010 |
| GQ995918 | Koblmüller et al. 2010 |
| KF205352 | Van Steenberge et al. 2015 |
| KF205354 | Van Steenberge et al. 2015 |
| KF205356 | Van Steenberge et al. 2015 |
| KF205358 | Van Steenberge et al. 2015 |
| *Kigoma morph* |  |
| KF205344 | Van Steenberge et al. 2015 |
| KF205348 | Van Steenberge et al. 2015 |
| KF205350 | Van Steenberge et al. 2015 |

Breman FC, Loix S, Jordaens K, Snoeks J, Van Steenberge M. Testing the potential of DNA barcoding in vertebrate radiations: the case of the littoral cichlids (Pisces, Perciformes, Cichlidae) from Lake Tanganyika. Molecular ecology resources 2016;16(6):1455-1464.

Koblmüller S, Egger B, Sturmbauer C, Sefc KM. Rapid radiation, ancient incomplete lineage sorting and ancient hybridization in the endemic Lake Tanganyika cichlid tribe Tropheini. Mol Phylogenet Evol 2010;55:318-334.

Van Steenberge M, Vanhove MP, Breman FC, Snoeks J. Complex geographical variation patterns in *Tropheus duboisi* Marlier, 1959 (Perciformes, Cichlidae) from Lake Tanganyika. Hydrobiologia 2015;748(1):39-60.
