# Supplementary material for: Comparative transcriptomics reveals candidate carotenoid color genes in an East African cichlid fish
Source: BMC Genomics. 2020 Jan 16;21:54. doi: 10.1186/s12864-020-6473-8 (PMC6966818; doi:10.1186/s12864-020-6473-8)
Supplement: Supplementary file 3 — Additional file 3: Table S2. Number of RNA sequencing reads obtained for each sample. Figure S1. HPLC chromatograms of skin extracts. Figure S2. Neutral lipid analysis by thin-layer chromatography (TLC). [file 12864_2020_6473_MOESM3_ESM.pdf]

Additional File 3

Comparative transcriptomics reveals candidate carotenoid color genes in an East African cichlid fish

Ehsan Pashay Ahi, Laurène A. Lecaudey, Angelika Ziegelbecker, Oliver Steiner, Ronald Glabonjat, Walter Goessler, Victoria Hois, Carina Wagner, Achim Lass, Kristina M. Sefc

Table S2. Number of RNA sequencing reads obtained for each sample

Sample	Raw PE reads	Quality trimmed PE reads
K-v1	6,880,727	6,848,376
K-v2	5,603,371	5,580,411
K-v3	5,138,573	5,108,611
K-v4	6,493,627	6,444,886
K-v5	8,712,352	8,662,280
K-d1	3,359,326	3,334,928
K-d2	6,098,774	6,072,158
K-d3	7,847,957	7,816,103
K-d4	7,981,010	7,938,947
K-d5	4,774,772	4,754,752
M-v1	12,431,473	12,375,228
M-v2	12,189,183	12,146,789
M-v3	8,105,338	8,738,421
M-v4	11,969,185	11,896,298
M-v5	8,772,517	8,738,421
M-d1	14,138,854	13,912,069
M-d2	10,918,296	10,813,557
M-d3	9,388,945	9,355,153
M-d4	12,768,511	12,709,420
M-d5	13,033,578	12,985,479

Figure S1: UV/VIS HPLC chromatograms of skin extracts. The panels compare chromatograms of extracts from yellow (A) and white skin regions (B) before (above) and after (below) saponification. Observed wavelength is 440 nm. Signals identified via comparison with carotenoid standards are labeled. Two unidentified high-abundant peaks in the yellow skin extract are labeled with mass and predicted carotenoid formulas. Mass differences to the theoretical m/z were <5 ppm. ‘e’ indicates putative carotenoid esters.

(A) Yellow-colored skin (0.47 mg wet weight of skin / µl acetone)

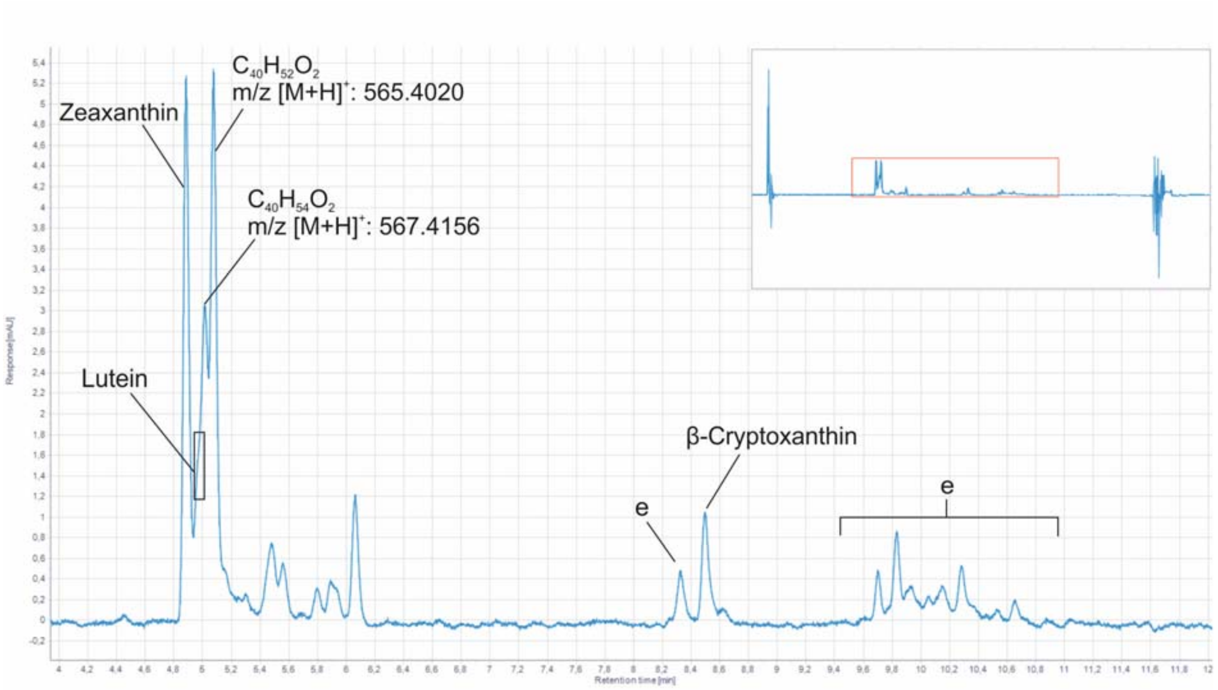

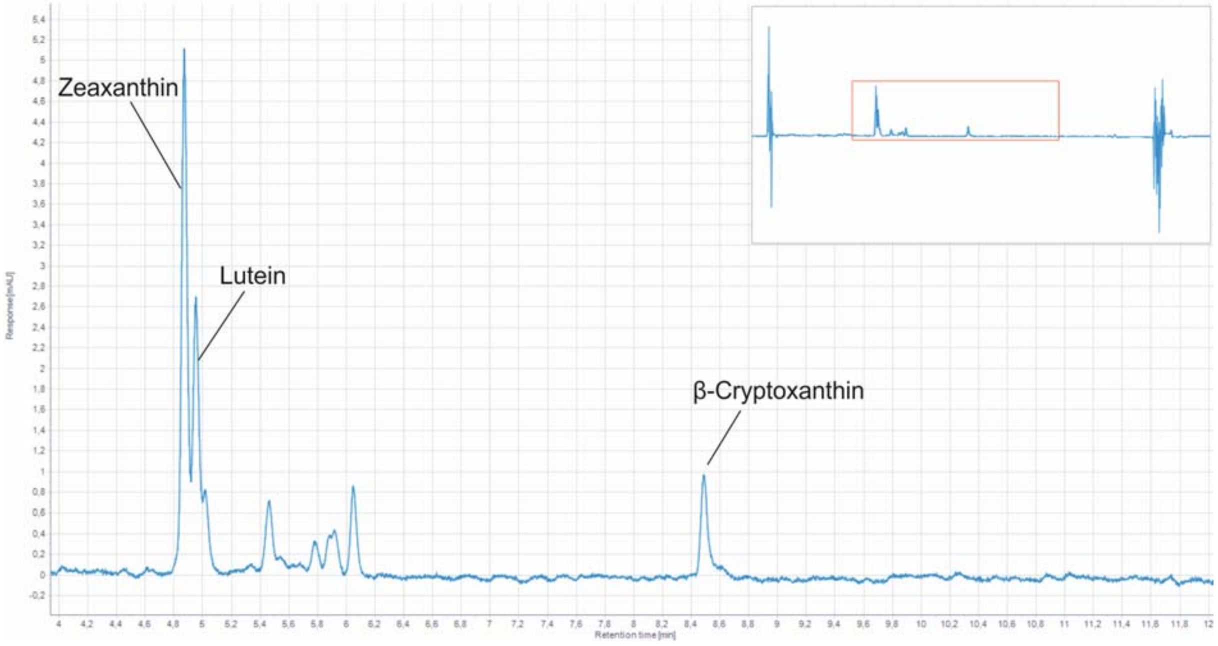

White-colored skin (0.21 mg wet weight of skin / µl acetone)

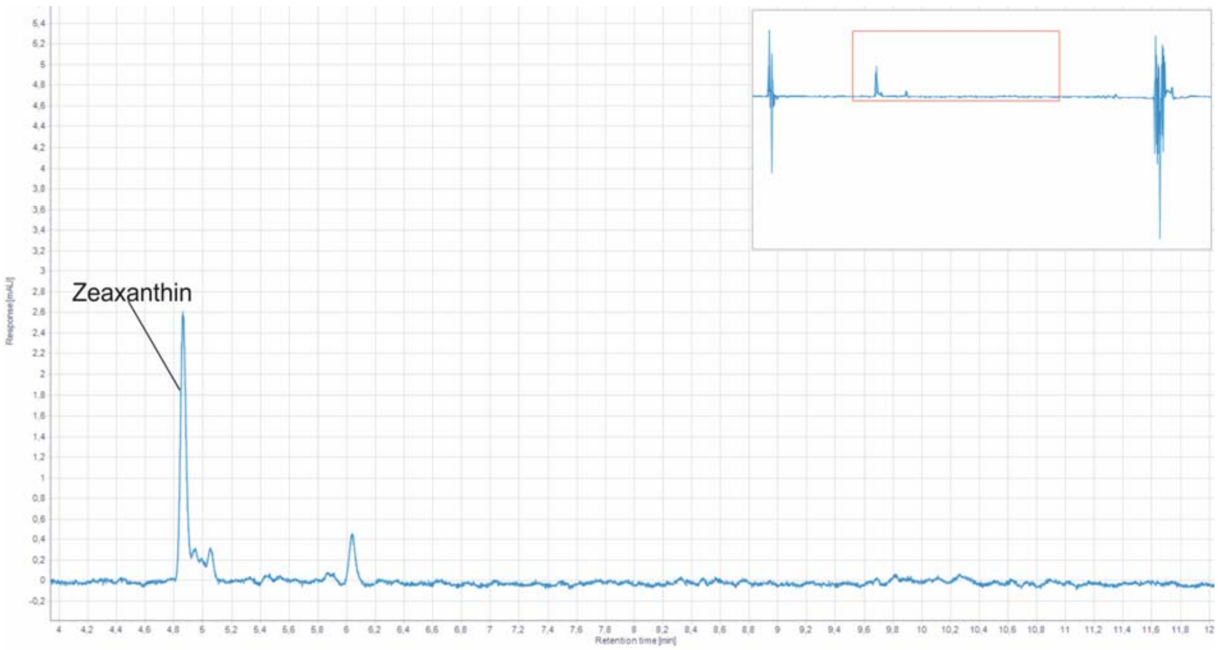

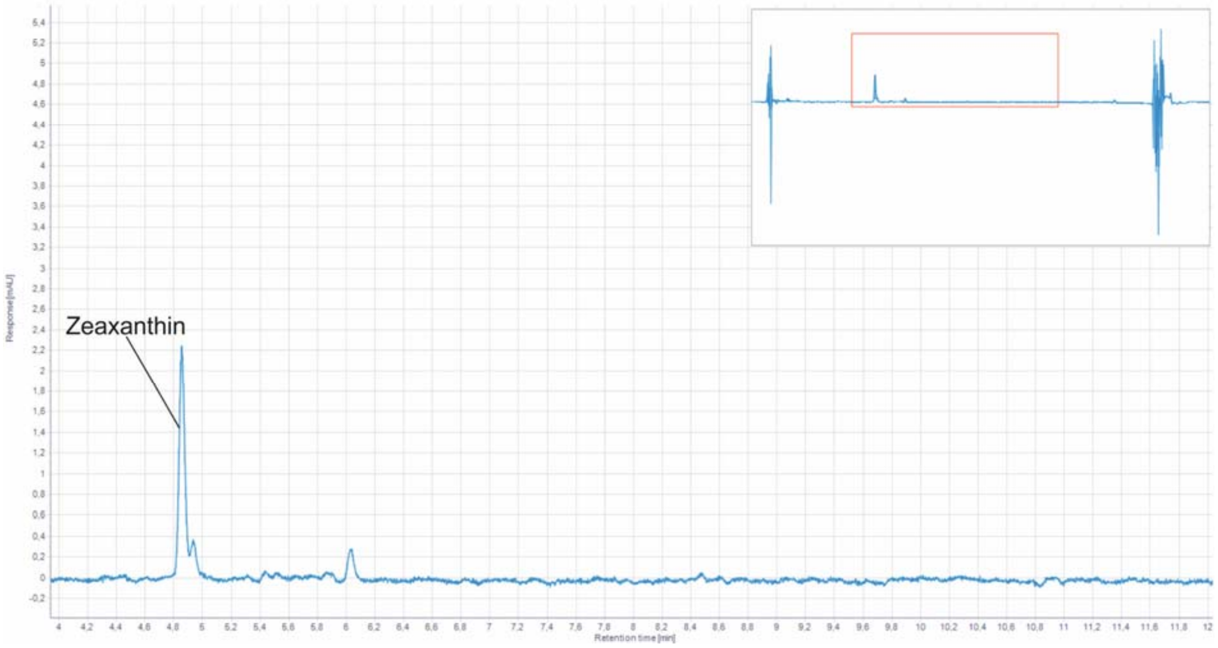

Figure S2: Neutral lipid analysis by thin-layer chromatography (TLC). Acetone lipid extracts of dorsal and ventral skin samples from six *T. duboisi* “Maswa” were spotted on a silica gel 60 and compared against standard solutions containing triolein or cholesterol. Chol, cholesterol; TG, triglycerides

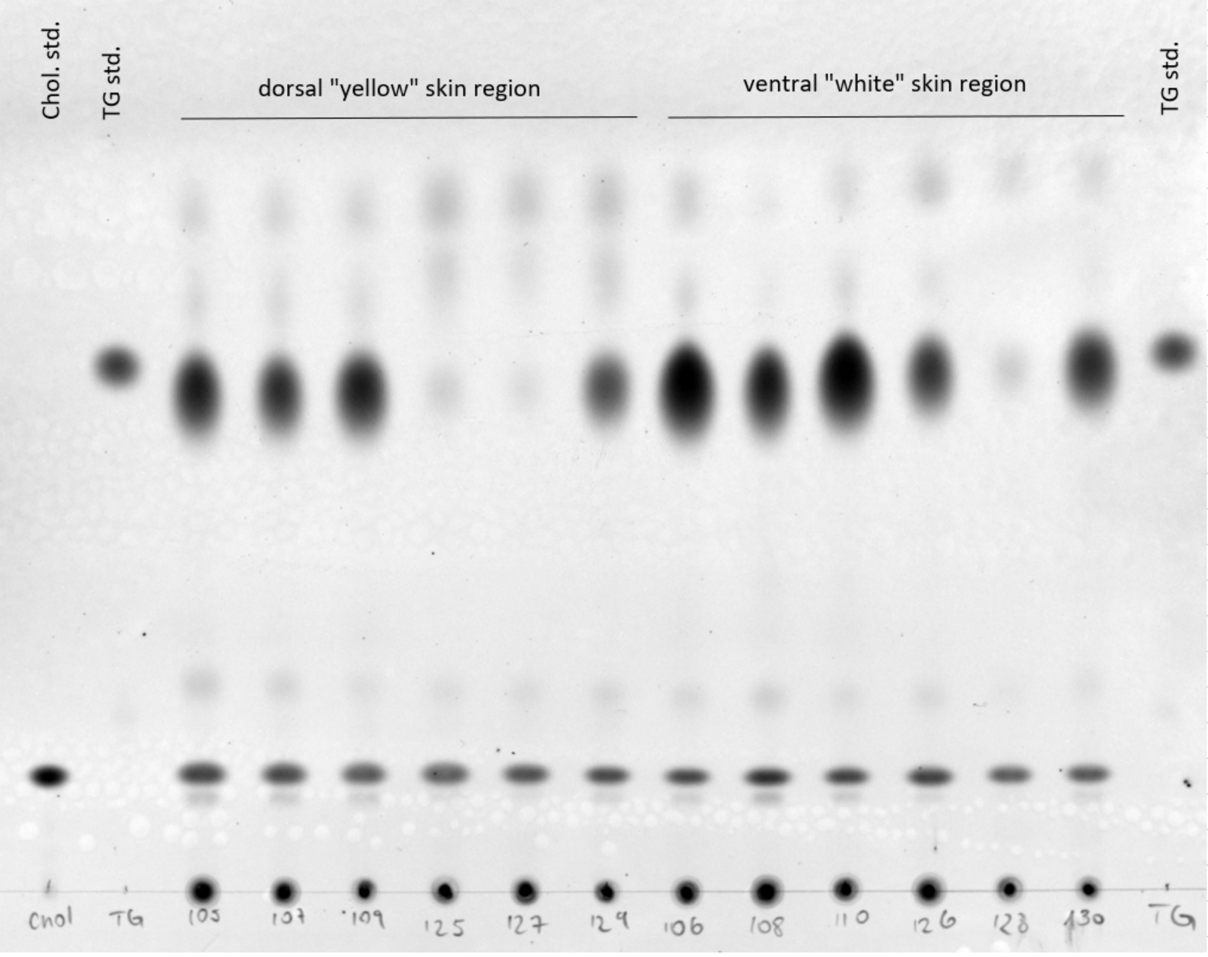

Fisch ID: 1 2 3 4 5 6 1 2 3 4 5 6
